# Evaluating police drug diversion in England: protocol for a realist evaluation

**DOI:** 10.1186/s40352-023-00249-2

**Published:** 2023-11-16

**Authors:** Alex Stevens, Nadine Hendrie, Matthew Bacon, Steve Parrott, Mark Monaghan, Emma Williams, Dan Lewer, Amber Moore, Jenni Berlin, Jack Cunliffe, Paul Quinton

**Affiliations:** 1 University of Kent, Medway, UK; 2 University of Sheffield, Sheffield, UK; 3 University of York, York, UK; 4 Loughborough University, Loughborough, UK; 5 Open University, Milton Keynes, UK; 6 Bradford Institute for Health Research, Bradford, UK; 7 User Voice, London, UK; 8 College of Policing, London, UK

## Abstract

There is increasing international interest in the use of police drug diversion schemes that offer people suspected of minor drug-related offences an educative or therapeutic intervention as an alternative to criminalisation. While there have been randomised trials of some such schemes for their effects on reducing offending, with generally positive results, less is known about the health outcomes, and what works, for whom, in what circumstances and why. This protocol reports on a realist evaluation of police drug diversion in England that has been coproduced by a team of academic, policing, health, and service user partners. The overall study design combines a qualitative assessment of the implementation, contexts, mechanisms, moderators and outcomes of schemes in Durham, Thames Valley and the West Midlands with a quantitative, quasi-experimental analysis of administrative data on the effects of being exposed to the presence of police drug diversion on reoffending and health outcomes. These will be supplemented with analysis of the cost-consequences of the evaluated schemes, an analysis of the equity of their implementation and effects, and a realist synthesis of the various findings from these different methods.

**Supplementary Information:**

The online version contains supplementary material available at 10.1186/s40352-023-00249-2.

## Background

There is increasing interest in the use of police drug diversion (PDD) schemes. Following the review by Stevens et al. (2022), we define such schemes as alternatives to criminalisation for minor drug-related offences, including—but not limited to—simple possession for personal use. They are alternatives that provide people suspected of such offences with an educative or therapeutic intervention, rather than being processed through prosecution and conviction. Such diversion has a long history in English policing, going back at least to the 1930s,^1^ but its use for drug offences was not openly acknowledged until more recently. Several police forces in the UK and elsewhere are now operating such schemes (Shaw et al., 2022; Stevens et al., 2022). Their use is likely to expand as the government drives policy on ‘out-of-court disposals’ (OOCDs), as signalled in the White Paper on a *Smarter Approach to Sentencing* (MoJ, 2020) and by the subsequent Police, Crime, Sentencing and Courts Act 2022. OOCDs include a range of disposals available for use by the police in England and Wales which enable police officers to resolve cases without suspects being charged or prosecuted, of which PDD is one form (Shaw et al., 2022).

There have been randomised trials of some such schemes—including Turning Point in the West Midlands and Checkpoint in Durham—for their effects in reducing offending, with generally positive results (Neyroud, 2018; Weir et al., 2021, 2022). Less is known about the contexts and mechanisms of such programmes in the UK, and about their effects on outcomes other than offending—particularly health outcomes. People who use drugs are highly vulnerable to a range of health harms, not only from overdose, but also from chronic health conditions (Lewer et al., 2021). PDD schemes offer the opportunity to divert people towards drug treatment services that are effective in treating substance use disorders and preventing death (Ma et al., 2019; Stevens et al., 2022). To develop useful knowledge on the contexts, mechanisms, moderators and outcomes of PDD, we decided to carry out a realist evaluation of PDD schemes in England (Pawson & Tilley, 1998). Realist evaluation enables us to move beyond analysing what works in PDD to consider what works, for whom, in what circumstances and why. The work has been co-designed with our policing partners and with User Voice, a charity led by people with lived experience of adverse contact with criminal justice agencies. It is funded by the Cabinet Office Evaluation Accelerator Fund, which aims to build the evidence base on priority policy areas with a view to informing future government spending reviews.

We will focus on the process, outcomes, cost consequences, and equity effects of PDD schemes in Durham, Thames Valley and the West Midlands. Our process evaluation will include a specific focus on the experiences of drug-involved suspects who are exposed to PDD schemes, whether they take up the diversion opportunities or not, as well as the police officers/staff and partner agencies who implement PDD schemes. In assessing outcomes, we will use data already collected by the police, NHS, and drug treatment services to assess the impacts of PDD on crime, hospitalisations and engagement with drug treatment. We will also examine how equitable the effects of PDD are (e.g. by ethnicity and gender). We will use existing realist frameworks such as EMMIE (Johnson et al., 2015), VICTORE (Cooper et al., 2020), and Carroll et al.’s (2007) conceptual framework for implementation fidelity. EMMIE highlights the Effect, Mechanisms, Moderators, Implementation and Economics of particular interventions or families of programmes (Johnson et al., 2015). VICTORE is a complementary but more detailed, realist framework which sensitises us to examine Volition, Implementation, Contexts, Time, Outcomes, Rivalry and Emergence (Cooper et al., 2020). Carroll et al’s (2007) framework is also complementary. It includes examination of four aspects each of adherence to the plans for the intervention (content, coverage, frequency, duration) and moderators of fidelity (intervention complexity, facilitation strategies, quality of delivery, participant responsiveness), plus the identification of essential components of the intervention.

Our analysis is split into six work packages which will produce a realist synthesis (Jagosh, 2019; Pawson, 2002) and a final, revised programme theory by the end of the project in spring 2025. In the remainder of this protocol, we report on the setting and content of the evaluated PDD schemes, and the methods adopted for each of the work packages of our evaluation. In creating this protocol, we have used the items of the RAMESES II reporting standards for realist evaluation (Welch & Tricco, 2016; Wong et al., 2016), although we have made some changes to their order (e.g. the programme theory appears in the section on work package 1) and we report methods separately for each work package. We will also use these standards for reporting results, including main findings, and consideration of strengths, weaknesses and future directions.

## The evaluated PDD schemes

In collaboration with local stakeholders and based on Hoffmann et al.’s (2014) template for intervention description and replication (TIDieR) checklist, we have developed manuals which describe the operation of each of the PDD schemes in the three focus areas: Durham (Hendrie et al. (2023a), Thames Valley (Hendrie et al. (2023b) and the West Midlands (Hendrie et al. (2023c). Their common features are that they involve police contact with people who use drugs who are suspected of committing an offence (e.g. drug possession). We refer to this target group as ‘drug-involved suspects’. In each area, PDD involves diversion to group or individual sessions of drug education and advice instead of proceeding with prosecution for the suspected offence. People who are diverted may also be referred to other services (e.g. local drug treatment agencies) if needed. There are some differences between the three focus areas. In the West Midlands and Thames Valley, the interventions to which people are diverted are provided by third sector organisations and people generally receive online or face-to-face drug education sessions. Eligibility in these areas is confined to people suspected of simple drug possession (with no intent to supply). In Durham, the eligibility criteria include a wider range of offences. Here, the diversion takes the form of one-to-one meetings with staff employed by the police (known as ‘navigators’). These similarities and differences will be studied in our evaluation to examine the various mechanisms and outcomes which they trigger.

The overall study design is a mixed methods realist evaluation which combines a: qualitative assessment of the implementation, contexts, mechanisms, moderators and outcomes of the evaluated PDD schemes; with a quantitative, quasi-experimental analysis of administrative data on the effects of being exposed to the presence of PDD schemes on reoffending and health outcomes. These will be supplemented with analysis of the cost-consequences of the evaluated PDD schemes, an analysis of the equity of their implementation and effects, and a realist synthesis of the various findings from these different methods.

### Work package 1: development of intervention manuals and a theory of change

This part of our work is already complete. It involved running in-person workshops with stakeholders in each of the three focus areas, plus a national stakeholder workshop. Stakeholders included local police officers and managers, and the staff and managers of the agencies which provide the interventions to which drug-involved suspects are diverted, as well as representatives from User Voice and local partner agencies (for example, staff from local NHS liaison and diversion services and public health substance misuse lead officials).^2^ In total, these workshops involved over 70 stakeholders. Prior to the three area workshops, police staff and the agencies that deliver the interventions provided draft descriptions of the PDD schemes based on the TIDieR checklist (Hoffmann et al., 2014), as well as a draft version of the PDD theory of change. The area workshops discussed real life examples of how the schemes operate in practice. In these discussions, we clarified or further adapted the scheme descriptions previously provided. This created more detailed description of the schemes. We also shared vignettes of possible drug possession scenarios, which allowed police officers and other staff to provide operational insight into how they would or would not apply a diversion to a drug-involved suspect.

The revised scheme descriptions for all three focus areas were shared at a national stakeholder meeting. This event provided all stakeholders the opportunity to agree the revised theory of change and their area scheme description.

**Fig. 1:**
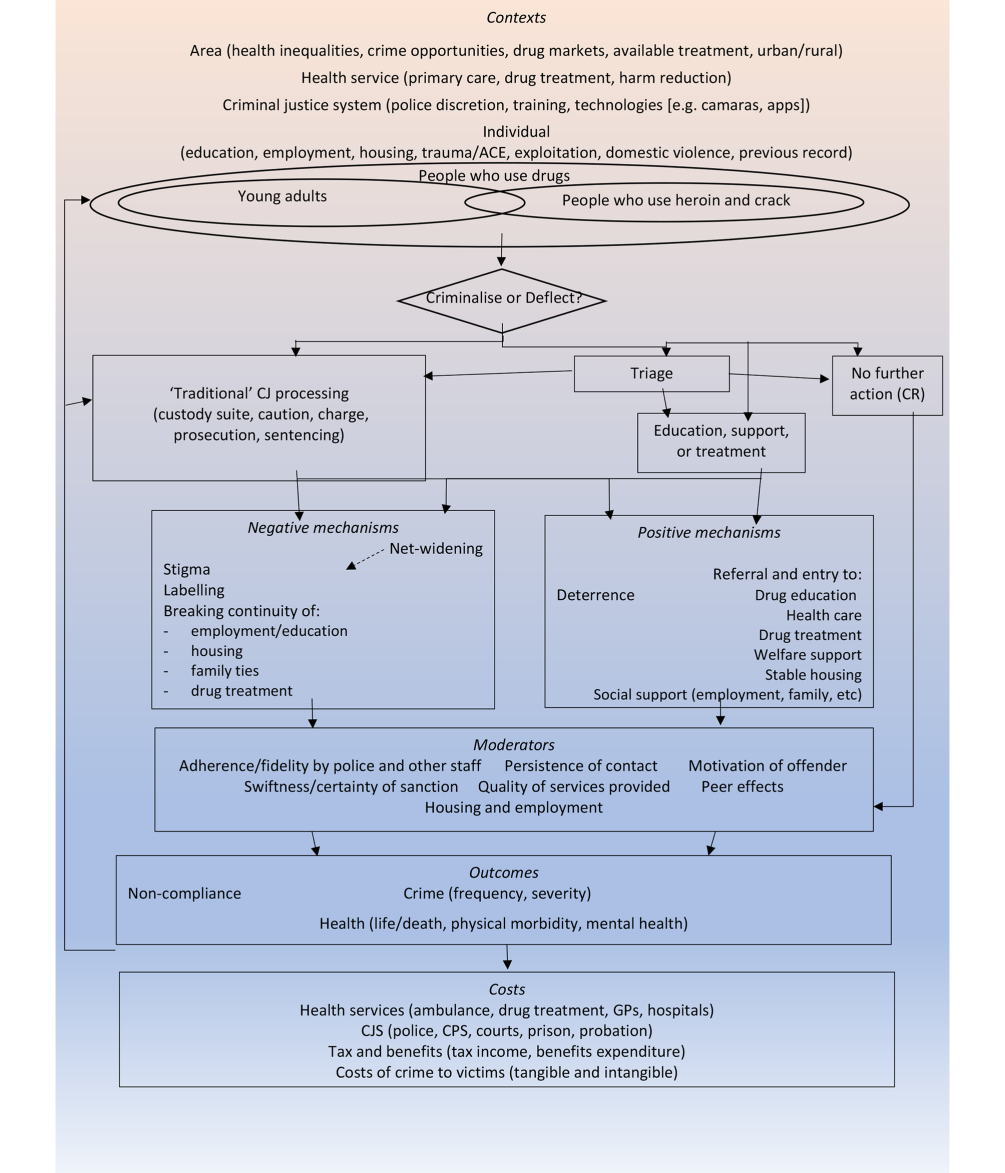
PDD theory of change

This theory of change was based on the programme theory provided by Stevens et al.’s (2022) realist review of alternatives to criminalisation for drug possession, with additional input from research published since the search was carried out for this review (in 2018). This additional research was found using similar search terms and databases to those used in the original review. The theory was further developed through discussion with stakeholders at the local and national workshops. The resulting theory of change is available online as a separate document (Stevens et al., 2023). In summary, it suggests that PDD may have positive effects on offending and health outcomes via two causal pathways. One is avoidance of the negative effects of criminalisation. The other is enhancing the support and treatment available to people who are at risk of drug-related offending and health harms. The theory of change also identifies a range of relevant contexts and moderators of the outcomes of PDD, as displayed in Fig. 1.

The theory of change and the three programme manuals (one each for Durham, Thames Valley and the West Midlands) provide the basis for our adaptive analysis of the process and effects of the three focus PDD schemes, as well as for the assessment of their implementation fidelity.

### Work package 2: process evaluation

Qualitative process evaluations will be undertaken in Durham, Thames Valley and the West Midlands to produce comparable information on the complex processes through which PDD schemes generate outcomes. This work package will investigate: (1) the implementation of PDD schemes; (2) whether and how PDD schemes produce change in drug-involved suspects; (3) the impact of contexts and moderators on how PDD schemes work; (4) perceptions of stakeholders (including drug-involved suspects) of multiple outcomes (including effects on reoffending, health, wellbeing, quality of life, stigmatisation, and family relations); and (5) fidelity of implementation of the PDD schemes.

Data will be collected through semi-structured interviews and focus groups with practitioners and drug-involved suspects. In total, we plan to do 225 interviews; 75 in each of the case study areas. Sample size will be determined by the achievement of data and inductive thematic saturation (Saunders et al., 2018). Focus groups will take place towards the end of the fieldwork period to discuss preliminary findings from interviews.

The inclusion criteria for practitioners is that research participants are involved in the strategy, management and/or delivery of PDD in Durham, Thames Valley or the West Midlands. Police participants will include senior officers in relevant leadership roles (e.g. on drugs and OOCDs), staff from the office of the Police and Crime Commissioner, managers of diversion schemes, and frontline officers. Purposive sampling will be used to recruit frontline officers from a range of roles (e.g. CID, response and neighbourhood teams) and policing districts, as well as to account for variables such as gender, ethnicity and length of service. Interviews will also be carried out with staff from the organisations that deliver the diversion programmes and other support services to which people who are diverted are referred (e.g. drug treatment, housing and employment).

Research participants who are drug-involved suspects will include: service users who were eligible for diversion, have been diverted, and are fully or partially participating in PDD schemes; and people who were eligible for diversion but were not diverted or are not participating. Interviews with drug-involved suspects will be carried out by User Voice’s peer researchers. Interviewees will be selected purposively to inform us on a range of experiences, according to gender, ethnicity, age, type of drug use (e.g. infrequent/frequent cannabis use, infrequent/frequent cocaine use, dependent use of heroin), and engagement in the diversion scheme (none/partial/full).

We will also gather aggregate police force level data for our three focus areas on the numbers of people who: are contacted as drug-involved suspects; are diverted to PDD schemes; participate in such schemes; complete the scheme; receive an OOCD; or receive some form of criminal justice sanction (e.g. caution, charge, sentence). We will ask for these data to be provided for each year from three years before the start of each PDD scheme to March 2024, and broken down by age group, gender and ethnicity. This will inform our analysis of implementation fidelity, as well as our equity assessment in work package 5.

Interviews and focus groups will be transcribed and anonymised and imported into Nvivo software for computer-assisted qualitative data analysis (Dalkin et al., 2021). We will also include memos on our descriptive analysis of the aggregate data in the qualitative dataset. Following adaptive theory (Layder, 1998), provisional codes for the analysis have been derived from our refined theory of change (see Fig. 1) and the conceptual frameworks we drew from EMMIE (Johnson et al., 2015), VICTORE (Cooper et al., 2020) and Carroll et al. (2007). Through a hybrid process of deductive and inductive reasoning and close reading of interview and focus group transcripts, as well as ‘attached memos’ (Dalkin et al., 2021), we will develop a final set of core and satellite thematic codes. These will form the basis for writing up findings on the contexts, mechanisms, moderators and outcomes of the three PDD schemes, and our analysis of the fidelity of implementation.

### Work package 3: quantitative outcome assessment

A quantitative outcome assessment will test hypotheses that the presence of PDD schemes in police force areas reduce adverse health and offending outcomes for drug-involved suspects. The analysis will involve comparing outcomes for cohorts of drug-involved suspects eligible for diversion in our three focus areas (i.e. police forces with PDD schemes), with those for cohorts of similar individuals in three comparison areas (i.e. forces without PDD schemes) that are in groups of police forces that are most comparable to the intervention forces (HMICFRS, 2023).

Participants in our quantitative analysis will be individuals who had contact with the police in relation to illicit drugs, who may have been eligible for diversion (regardless of whether diversion was available, offered or taken up by that individual). Each PDD and comparison police force will identify individuals who had contact with the police for a qualifying offence between 1 October and 2021 and 30 September 2022 and were aged at least 18 years. Qualifying offences will be (a) simple possession of any controlled drug (personal use), and (b) any of: shoplifting, assault, criminal damage, criminal damage without intent to commit, possession of drugs, drunk and disorderly, drunk and incapable, any theft (other than burglary); in combination with a suspected or proven offence in contravention of the Misuse of Drugs Act 1971 or the Psychoactive Substances Act 2016, in the preceding 3 years, OR a flag in police records for involvement with illicit drugs.

We will use two primary outcomes for health and reoffending:

1. Health—the count of hospital episodes (including unplanned/emergency inpatient admissions and emergency department visits) during the follow-up period that are related to drugs, alcohol, or accidents.
2. Reoffending—a count of how many offences participants commit in the follow-up period, as recorded in the PNC and adjusted for the time that each participant was in the follow-up period.

We will also use the following secondary outcomes:

1. Health—the measures will be (a) any entry into structured treatment for drug or alcohol use in a community setting during the follow-up period; and (b) retention in treatment for at least 28 days for those who start drug or alcohol treatment.
2. Reoffending—(a) a dichotomous measure of any offending, with analysis offset by the duration of follow-up, and (b) a measure of the total social cost of these crimes, calculated using the costs attributed to each type of crime according to Home Office estimates of the social costs of each type of crime (Heeks et al., 2018).

The data for the quantitative impact assessment will be compiled using individual-level data linkage between databases of administrative records. Participating police forces will share lists of eligible individuals with the Department of Health and Social Care (DHSC), which is a partner in the project. The DHSC will link the data to four national health and criminal justice databases, and then pseudonymise the data before analysis within a secure data system. The four national databases will be:

1. The Police National Computer, which will be used to derive a measure of historical offending at study entry, and the primary and secondary reoffending outcomes;
2. The National Drug Treatment Monitoring System, which will be used to measure secondary health outcomes (i.e. entry to and retention in drug and alcohol treatment services);
3. Hospital Episode Statistics, which will be used to measure our primary health outcome (i.e. hospital episodes) (Herbert et al., 2017).
4. The Office for National Statistics mortality database, which we will use to identify participants who die during the follow-up period and are to be excluded from our analysis. Deaths will only be used to censor follow-up and not as an outcome because their relative rarity would result in a lack of statistical power for detecting significant effects.

We plan to use multivariable regression to estimate the effect of PDD on primary and secondary outcomes. Our analysis will control for potential confounders including age, sex/gender, ethnicity, health, previous experience of drug or alcohol treatment, offending history, reason for police contact, date of cohort entry, and the force-level baseline probability of each outcome. Our primary outcomes are count variables, and we will therefore use a mixed negative binomial model offset by the logged duration between the study entry date and the end of follow-up. We plan to use a negative binomial model rather than a Poisson model because we anticipate that both outcomes will be overdispersed, with some participants having high counts.

In our primary analysis, we will compare outcomes for all drug-involved suspects eligible for diversion in our focus areas (regardless of whether they were offered or took up diversion) with those for similar suspects in control areas. This is akin to an ‘intention to treat’ design in an experimental study (Hernán & Robins, 2016).

In our secondary analyses, we will:

1. Compare outcomes for participants who are diverted with those who are not. This is comparable to a ‘per protocol’ analysis in experimental trials (Hernán & Robins, 2016);
2. Report access to diversion by age, sex, ethnicity, and socioeconomic status (as a complement to work package 5 on equity of effects).
3. Test whether the effectiveness of diversion for each outcome varies by police force (while acknowledging that this analysis will have lower statistical power).

### Work package 4: cost-consequence analysis

In order to evaluate the economic effects of PDD, a cost-consequences analysis (CCA) framework will be conducted based on the various perspectives of the stakeholders. This disaggregated analysis enables the costs and consequences for each individual budget-holder to be clearly defined and presented and their own budgetary impact delineated. In this study, these stakeholders include local police forces, local health services, and members of the public.

The CCA will estimate the costs of providing the PDD intervention. We will record the quantities of resources used in each intervention area and apply local unit costs. Initial information on resource use has been collected from policing partners and local service providers alongside the TIDieR information collected for work package 1. Additional information on staff time, staffing grade, other specific resources used within the programme and overheads will be collected at the end of work package 2. Local unit costs will be applied to resource quantities to derive the total cost of providing the intervention and the cost per participant. Intervention costs will be presented for each area which will be anonymised for reporting purposes.

The CCA builds on the primary and secondary outcomes outlined in work package 3. We will estimate the treatment costs incurred by the NHS based on conditions plausibly affected by the intervention. Costs will be derived by applying national average costs to the cases identified using the NHS National Cost Collection database of unit costs (NHS England, 2022).

The social and economic costs associated with reoffending (e.g. arrests, convictions and imprisonments) as quantified in work package 3 will be included in the CCA. We will apply unit costs for each criminal justice event recorded in the data linkage. Treasury approved methods will underpin the CCA and unit costs will be extracted from the latest version of the Greater Manchester Combined Authority Unit Cost Database and the Home Office estimates of the costs of crime (GMCA Research Team, 2021; Heeks et al., 2018). This will cover the costs of crime, health and social services. Costs of crime will be presented in terms of fiscal costs and cost to society. The domains for the CCA for the 12 months following police contact are the cost of hospital admissions relating to acute mental health, alcohol and other drugs, and the criminal justice system costs of criminal conviction.

Costs will be presented in a disaggregated format which will provide clear information to the different stakeholders with respect to their financial impacts. The CCA will estimate the longer-term discounted costs over the next five years based on criminal justice contacts and health care episodes. We will construct a hybrid decision tree and Markov model based upon drug use status following PDD. The model uses the rates of post-PDD drug use, all cause death rates, and reconviction rates as transition probabilities. A mean patient cost for health care and criminal justice in each of the Markov states is applied and the simulation will be run for a period of five years. Costs and outcomes occurring beyond one year are discounted using a discount rate of 3.5% as recommended by the National Institute for Health and Clinical Excellence (NICE, 2020). We will use the NHS National Costs Collection and GMCA database combined with data from work package 3 and parameters from the published literature to estimate the return on investment. The costs of the PDD programme will also be scaled up to national level in order to estimate the overall cost of providing the intervention it if were to be nationally implemented.

### Work package 5: equity assessment

In the light of concerns over health inequalities and ethnically disproportionate policing (Shiner et al., 2018), we will carry out an assessment of the equity aspects of our focus PDD schemes. This work package will draw on data collected for work packages two and three, and so will have the same participants. Quantitatively, we will test the hypotheses that there are significant differences in the proportion of eligible suspects that are offered diversion away from criminalisation by gender and ethnicity. We will also test the hypotheses that there are similar differences among suspects who are offered diversion in terms of: attending diversion; completing it successfully; and ending up with OOCDs or criminal sanctions for suspected offences. This will enable us to assess whether PDD leads to ‘net-widening’ and ‘mesh-thinning’ in these areas. These two phenomena, originally identified by Cohen (1979), refer to processes whereby purported alternatives to criminalisation can actually bring more people into the net of penal control and increase the obligations placed upon them. Given the known disproportionality of drug law enforcement in England and Wales (Shiner et al., 2018; Stevens, 2011), it is possible that these processes have more of an impact on people who are racialised as Black. We will seek to examine this possibility in both the aggregate data, and in the individual-level data collected for Work Package 3, although we are not yet certain that the individual-level data will include information on ethnicity to do so.

Using the qualitative data collected for the second work package, we will also look for any differences in the processes and reported outcomes of PDD schemes for people with other protected and PROGRESS + characteristics (O’Neill et al., 2014). These include place of residence, race/ethnicity, occupation/class, gender/sex, religion, education, socio-economic status, social capital, age, disability, time-dependent characteristics (e.g. educational transitions, leaving home), and relevant personal circumstances (e.g. previous experiences of local authority care and contact with the criminal justice system).

We have included questions in our schedules for interviews and focus groups and codes in our provisional coding framework that will elicit information on these potential axes of disparity and discrimination. In our qualitative analysis, we will run queries that search for data on these issues. This will enable us to provide distinct qualitative data that supplements the quantitative analysis of equity. Unfortunately, the quantitative datasets to which we will have access do not provide data on the full range of PROGRESS + characteristics.

### Work package 6: realist synthesis

In this final work package, we will bring together all our findings in the form of a realist synthesis of what works, for whom, in what circumstances and why. To do this we will employ the EMMIE and VICTORE framework (Cooper et al., 2020; Johnson et al., 2015). Taken together, the work packages provide detail on all the components of these framework.

Effect is synonymous with outcomes and so data from work packages 3 (quantitative outcome assessment) and 5 (equity assessment) will be used here. The Mechanisms, Moderators and Implementation are central to the delivery of the process evaluation in work package 2. Work package 4 will detail the economics of PDD in the form of the CCA. Our analysis in work package 2 also employs the VICTORE framework to sensitise us to issues of Volition (the intentions and choices of people involved in the PDD schemes), Time (including changes in implementation over time), Rivalry (the presence and effects of similar or competing interventions for the target group), and Emergence (the features of PDD schemes that arise from the combinations of other factors). Work packages 2 and 5 will provide detail on the implementation of PDD schemes. Work package 4 will detail the economics of PDD in the form of the CCA.

As realism encourages us to think beyond ‘what works’, in our synthesis we will unpack what it is about PDD that leads to the observed pattern of outcomes. In other words, we do not view exposure to PDD as an independent variable that can be experimentally closed off from all other factors (Deaton & Cartwright, 2018). We are interested in what it is about PDD schemes that produce outcomes in particular contexts. In this sense, the findings of the realist synthesis will enable us to refine further the programme theory in Fig. 1 and then use this as the platform for developing actionable guidance for the future rollout of PDD programmes.

## Dissemination

We have developed a dissemination plan to reflect the diverse needs and interests of the likely audiences for the PDD evaluation. These audiences are varied and consist of: (a) police and health partners (such as government departments, police forces, local authorities and service providers); (b) public stakeholders (e.g. criminal justice charities and advocacy groups); (c) service users; (d) academics and other researchers; and (e) the wider general public.

Dissemination of our research findings will be principally based around two types of publication. First, a series of academic journal articles covering different aspects of the PDD evaluation are to be submitted for publication. Separate articles are planned on the results of each of the work packages (i.e., the qualitative process evaluation, quantitative impact assessment, cost-consequence analysis, equity assessment, and realist synthesis). In addition, we will consider submitting articles on a range of thematic, theoretical and methodological issues (such as data linkage, policy learning, and research coproduction). The main audiences for this type of publication will be academics, researchers, and a range of partners and stakeholders. To ensure transparency and maximise the public use and social value of the research, we plan to make preprint and open access copies of these publications available via the Kent Academic Repository.

Second, the College of Policing, which is one of the partners in the project, will publish a summary of our evaluation findings. This summary may take the form of a single report covering the evaluation as a whole or a series of reports, one for the results of each work package. The summary report(s) will be written in plain English for non-specialist audiences (such as the police, public and some stakeholders), and will be made publicly available under an open licence via the National Police Library. The College of Policing also plans to host links to all journal articles and plain-English summaries on its website.

In addition to these formal publications, we intend to disseminate the PDD evaluation findings via a range of more informal communication channels. These channels are likely to include: briefings to relevant government departments and agencies, and the media; the circulation of evaluation outputs through national and regional networks (such as the networks of the members of our project advisory group that extend across the UK civil service) and online knowledge sharing platforms (like the Police Knowledge Hub); conference presentations to policy, practitioner and academic audiences; local workshops with service users and practitioners; social media; and specialist and sector-specific publications.

Pathways to research impact on policy and practice are built into our dissemination plan by virtue of the evaluation being funded by the Cabinet Office, and the College of Policing and National Police Chiefs’ Council (NPCC) being collaboration partners. The Cabinet Office’s Evaluation Accelerator Fund that has financed the PDD evaluation was established for “the creation of actionable evidence in [Government] priority areas to inform decision-making at the next spending review” (HM Government, 2023). The reports we are required to submit to the Cabinet Office as part of the funding arrangement may, therefore, be used to support business cases to the Treasury for an increase in government spending on PDD (if the evaluation can demonstrate that PDD has a positive impact on outcomes and is cost-effective). Collaboration with the College of Policing and NPCC provides us with opportunities to influence policing policy and practice. The College of Policing—as the professional body for policing in England Wales—sets the standards for policing in the form of the national policing curriculum and operational guidance. The College of Policing has agreed to develop evidence-based guidelines on the implementation of PDD, subject to the evaluation showing that it had a positive impact on outcomes. The NPCC sets the strategic direction for policing and is responsible for coordinating policing activities across England & Wales, and, as such, will have an important role in supporting implementation of the College’s guidance.

## Discussion

There is a particular need for more information on the health outcomes of PDD schemes, and of the contexts, mechanisms and moderators of how they produce effects on health and offending. This study will provide such information in ways that will assist the future commissioning and delivery of such schemes. This is especially important, given that expansion of such schemes in the UK is likely following the inclusion of diversion in the UK’s ten-year drug strategy (HM Government, 2021) and the decision by the Scottish government to expand the use of diversion schemes (Scottish Government, 2023), despite some concerns that diversion is adding to rather than replacing the burdens of criminalisation (Price et al., 2021). There is also international interest in such diversion schemes, as shown by the rapid expansion of programmes such as Law Enforcement Assisted Diversion in the USA from its origins in Seattle (Collins et al., 2017). This study will meet the national and international need for more and better research on the processes and outcomes of police drug diversion.

### Electronic supplementary material

Below is the link to the electronic supplementary material.

Supplementary Material 1

Supplementary Material 2

## Data Availability

In order to protect the confidentiality of participants, we have promised ethical reviewers of this study that the original data will not be publicly released, due to the small but important risk that these data could be used to identify individual participants.

## Footnotes

1 A letter to the Metropolitan Police from a Home Office official, dated 11th September 1934, stated, ‘in many cases the threat of proceedings has been used as a lever for compelling them [“drug addicts”] to submit themselves to some kind of treatment or control’ without prosecution actually occurring (Whitelegg, 1934). Such informal diversion was still in operation in the 1960s for ‘registered addicts’ (Weisman, 1972, p. 64).

2 In England, liaison and diversion schemes support people who have mental health, learning disability, substance misuse or other vulnerabilities who come into contact with the police. The treatment of substance use disorders is funded through local government public health directorates.

## Abbreviations

ACMD: Advisory Council on the Misuse of Drugs
CCA: Cost consequence analysis
CID: Criminal Investigation Department
DHSC: Department of Health and Social Care
EMMIE: Effect, mechanisms, moderators, implementation, economics
GMCA: Greater Manchester Combined Authority
HMICFRS: His Majesty’s Inspectorate of Constabulary, Fire and Rescue Services
MoJ: Ministry of Justice
NHS: National Health Service
NICE: National Institute for Health and Clinical Excellence
NPCC: National Police Chiefs Council
OOCD: Out of court disposal
PDD: Police drug diversion
PROGRESS: Race/ethnicity/culture/language, occupation, gender/sex, religion, education, socioeconomic status, social capital
TIDieR: Template for intervention description and replication
VICTORE: Volition, implementation, contexts, time, outcomes, rivalry, economy
