# Correction: Evaluating police drug diversion in England: protocol for a realist evaluation

**DOI:** 10.1186/s40352-023-00255-4

**Published:** 2023-12-19

**Authors:** Alex Stevens, Nadine Hendrie, Matthew Bacon, Steve Parrott, Mark Monaghan, Emma Williams, Dan Lewer, Amber Moore, Jenni Berlin, Jack Cunliffe, Paul Quinton

**Affiliations:** 1 University of Kent, Medway, UK; 2 University of Sheffield, Sheffield, UK; 3 University of York, York, UK; 4 Loughborough University, Loughborough, UK; 5 Open University, Milton Keynes, UK; 6 Bradford Institute for Health Research, Bradford, UK; 7 User Voice, London, UK; 8 College of Policing, London, UK

**Correction:** ***Health Justice*** **11, 46 (2023)**

10.1186/s40352-023-00249-2

Following publication of the original article (Stevens et al., 2023), a mistake in the reference citation of Hendrie et al. (2023a, 2023b, 2023c) found on the first paragraph under “**The evaluated PDD schemes”** section has been corrected.

The correct line should be:

Durham (Hendrie et al. (2023a), Thames Valley (Hendrie et al. (2023b) and the West Midlands (Hendrie et al. (2023c).

The original article (Stevens et al., 2023) has been updated.
