# Supplementary material for: Evaluating police drug diversion in England: protocol for a realist evaluation
Source: Health Justice. 2023 Nov 16;11:46. doi: 10.1186/s40352-023-00249-2 (PMC10652635; doi:10.1186/s40352-023-00249-2)
Supplement: Supplementary file 2 — Supplementary Material 2 [file 40352_2023_249_MOESM2_ESM.docx]

Cabinet Office

70 Whitehall

SW1 2AS

| Professor Alex Stevens  University of Kent  Giles Lane  Canterbury CT2 7NZ  11th August 2022 |  |
| --- | --- |

Dear Alex,

**Grant Scheme: Evaluation Accelerator Fund**

**GGIS scheme number G2-SCH-2022-07-11481**

**Programme: Police drug diversion (PDD): A realist impact, process and economic evaluation**

**Reference: EAF-COP-PDD**

Cabinet Office (the **Authority**) is pleased to offer you a Grant for the performance of the research programme *Police Drug Diversion (PDD): A realist impact, process and economic evaluation,* up to the Maximum Sum of £1,802,233, subject to your agreement to, and compliance with, the terms and conditions set out in this Grant Funding Letter.

The funding settlement (the “Grant”) is for a total of £1,802,233 as follows:

- £429,537 in 2022/23
- £704,375 in 2023/24
- £668,321 in 2024/25

The grant for the financial year commencing 1^st^ April 2022, is made by the Rt Hon Jacob Rees Mogg MP, under the statutory authority provided by section 70 of the Charities Act 2006. The University of Kent is an exempt charity subject to the provisions of the Charities Act 2011 (HMRC reference number XN5452).

The Grant must only be used for activities comprised in (or necessary for, or incidental to) the following Project: *Police Drug Diversion (PDD): A realist impact, process and economic evaluation*. The key aims, objectives, outputs and outcomes are detailed in the Grant Agreement dated 11th August 2022.

**Payment**

The grant will be paid quarterly in arrears as detailed in the payment schedule in the Grant Agreement dated August 11th 2022. Cabinet Office will pay the grant monies only to the organisation to which this offer is made. The Cabinet Office will hold you responsible for the grant, including any portion of that, which you forward to the other partners. You will need to ensure, therefore, that they spend the money appropriately and within the conditions of this offer.

This grant agreement will expire on 31/03/2025. All grant funding received should be fully spent, or committed to be spent, by this date.

**Contact Points and Questions**

The person monitoring your work and responsible for monitoring your compliance with our grant conditions, will be Levin Wheller (Evaluation Lead, Evaluation Task Force). Steven Inglese in the Cabinet Office Central Financial Management Team will be responsible for paying you the grant.

Please feel free to contact Levin Wheller ([levin.wheller@cabinetoffice.gov.uk](mailto:levin.wheller@cabinetoffice.gov.uk)) or the Evaluation Accelerator Fund mailbox ([eaf@cabinetoffice.gov.uk](mailto:eaf@cabinetoffice.gov.uk)) directly should you have any questions about any of the matters mentioned in this letter.

To signify your acceptance of this offer you should **email** your acceptance back and have one copy of this letter signed (overleaf) and returned to me.

Yours sincerely

Levin Wheller,

Evaluation Lead,

Evaluation Task Force

Cabinet Office

We have read and understand all the terms and conditions of this offer and we formally accept them on behalf of

The University of Kent

………………………………………………………………………………………

*[insert name of organisation]*

Signed (Director/Chief Executive or equivalent, University of Kent)

..............................…..........

Name .............................................

Date .............................................

Signed (Director/Chief Executive or equivalent, Cabinet Office)

..............................…..........

Name .............................................

Date ............................................
