# Supplementary material for: Evaluating police drug diversion in England: protocol for a realist evaluation
Source: Health Justice. 2023 Nov 16;11:46. doi: 10.1186/s40352-023-00249-2 (PMC10652635; doi:10.1186/s40352-023-00249-2)
Supplement: Supplementary file 1 — Supplementary Material 1 [file 40352_2023_249_MOESM1_ESM.pdf]

University of Kent

Division for the Study of Law, Society and Social Justice

07/03/2023
Application Ref: 0780b

Dear Professor Stevens,

SRC Ethical Approval for "Police Drug Diversion (PDD): A Realist Impact, Process and Economic Evaluation "

Your research project has now been granted ethical approval by the Staff Review Committee. You are now able to commence your research.

It is essential that we keep our records up to date. In order to achieve this, please send us some feedback with regard to your project. For example, please notify us when your project has been completed, whether or not it was successful, and let us know about any ethical problems or questions that have arisen during the project.

Yours sincerely,

KJones

Professor Karen Jones
Chair
Staff Review Committee
